# Índice Hemodinâmico Agudo Prediz Mortalidade Intra-Hospitalar de Pacientes com Insuficiência Cardíaca Aguda Descompensada

**DOI:** 10.36660/abc.20190439

**Published:** 2021-01-27

**Authors:** Renata R. T. Castro, Luka Lechnewski, Alan Homero, Denilson Campos de Albuquerque, Luis Eduardo Rohde, Dirceu Almeida, João David, Salvador Rassi, Fernando Bacal, Edimar Bocchi, Lidia Moura

**Affiliations:** 1 Brigham and Womens Hospital Boston EUA Brigham and Womens Hospital – Medicine, Boston - EUA; 2 Hospital Naval Marcilio Dias Rio de JaneiroRJ Brasil Hospital Naval Marcilio Dias, Rio de Janeiro, RJ - Brasil; 3 Faculdade de Medicina Universidade Iguaçu Nova IguaçuRJ Brasil Faculdade de Medicina, Universidade Iguaçu, Nova Iguaçu, RJ - Brasil; 4 Pontifícia Universidade Católica do Paraná CuritibaPR Brasil Pontifícia Universidade Católica do Paraná, Curitiba, PR - Brasil; 5 Universidade Estadual do Rio de Janeiro Rio de JaneiroRJ Brasil Universidade Estadual do Rio de Janeiro, Rio de Janeiro, RJ - Brasil; 6 Universidade Federal do Rio Grande do Sul Porto AlegreRS Brasil Universidade Federal do Rio Grande do Sul, Porto Alegre, RS - Brasil; 7 Universidade Federal de São Paulo São PauloSP Brasil Universidade Federal de São Paulo, São Paulo, SP – Brasil; 8 Hospital de Messejana FortalezaCE Brasil Hospital de Messejana, Fortaleza, CE - Brasil; 9 Universidade Federal de Goiás GoiâniaGO Brasil Universidade Federal de Goiás, Goiânia, GO - Brasil; 10 Universidade de São Paulo Instituto do Coração São PauloSP Brasil Universidade de São Paulo Instituto do Coração, São Paulo, SP – Brasil

**Keywords:** Insuficiência Cardíaca, Frequência Cardíaca, Pressão Arterial, Prognóstico, Mortalidade

## Abstract

**Fundamento:**

O exame físico permite a avaliação prognóstica de pacientes com insuficiência cardíaca (IC) descompensada, porém não é suficientemente confiável e depende da experiência clínica do profissional. Considerando as respostas hemodinâmicas a situações do tipo “luta ou fuga” tais como a admissão no serviço de emergência, foi proposto o índice hemodinâmico agudo (IHA), calculado a partir da frequência cardíaca e pressão de pulso.

**Objetivo:**

avaliar a capacidade prognóstica intra-hospitalar do IHA na IC descompensada.

**Métodos:**

estudo prospectivo, multicêntrico e observacional baseado no registro BREATHE, incluindo dados de hospitais públicos e privados no Brasil. Foram utilizadas análises ROC ( *Receiver Operating Characteristic* ), de estatística c e de regressão multivariada, assim como o critério de informação de Akaike, para testar a capacidade prognóstica do IHA. O valor-p < 0,05 foi considerado estatisticamente significativo.

**Resultados:**

Foram analisados dados de 463 pacientes com IC com fração de ejeção reduzida a partir do registro BREATHE. A mortalidade intra-hospitalar foi de 9%. A mediana do IHA foi considerada o valor de corte (4 mmHg⋅bpm). Um baixo IHA (≤ 4 mmHg⋅bpm) foi encontrado em 80% dos pacientes falecidos. O risco de mortalidade intra-hospitalar em pacientes com baixo IHA foi 2,5 vezes maior que aquele para pacientes com IHA > 4 mmHg⋅bpm. O IHA foi capaz de predizer independentemente a mortalidade intra-hospitalar na IC aguda descompensada [sensibilidade: 0,786; especificidade: 0,429; AUC (área sob a curva): 0,607 (0,540-0,674), p = 0,010] mesmo depois dos ajustes para comorbidades e uso de medicamentos [razão de chances (RC): 0,061 (0,007-0,114), p = 0,025].

**Conclusões:**

O IHA é capaz de predizer independentemente a mortalidade intra-hospitalar na IC aguda descompensada. Esse índice simples e realizado à beira do leito pode se mostrar útil em serviços de emergência. (Arq Bras Cardiol. 2021; 116(1):77-86)

## Introdução

A insuficiência cardíaca (IC) é uma das principais razões de admissões hospitalares de emergência no mundo ocidental.^1^ Apesar de estudos terem mostrado que o tratamento com um especialista em IC pode fornecer melhores resultados, a maioria dos casos de descompensação aguda da IC são originalmente avaliados e manejados por médicos emergencistas^2 , 3^ em instituições com diferentes disponibilidades de recursos.

Mesmo com o avanço da tecnologia e dos dispositivos médicos, o exame físico continua sendo o pilar principal da avaliação de pacientes com IC.^4 , 5^ Médicos avaliam a congestão e perfusão a partir do histórico do paciente e do exame físico, determinando perfis hemodinâmicos que podem guiar a terapêutica e fornecer informações prognósticas numa situação de IC aguda.^6^ Apesar da praticidade, a avaliação da perfusão feita pelo médico não é suficientemente confiável^7^ e depende da experiência do profissional,^8 , 9^ posto que fornece informações subjetivas.^10^ Logo, parâmetros prognósticos objetivos que podem ser facilmente obtidos em um atendimento de emergência seriam úteis no manejo da IC aguda.

A pressão arterial e a frequência cardíaca são parâmetros que podem ser facilmente obtidos por qualquer profissional de saúde, com boa reprodutibilidade e acurácia.^11 , 12^ A pressão arterial sistólica é um preditor independente de desfechos intra-hospitalares e pós-alta na IC aguda.^13 , 14^ Além disso, baixos valores de pressão arterial e pressão de pulso proporcional são indicadores de baixa perfusão.^4 , 6 , 9^

A relação entre frequência cardíaca em repouso na admissão e o prognóstico de pacientes com IC não é completamente direta. Na realidade, a literatura apresenta resultados controversos, indicando que uma frequência cardíaca alta no momento da admissão pode estar relacionada a um prognóstico melhor ou pior.^15 - 17^ Apesar de baixas frequências cardíacas em repouso indicarem um menor risco em pacientes com IC crônica estável com fração de ejeção reduzida,^18 , 19^ é inegável que a capacidade de aumentar a frequência cardíaca numa reação do tipo “luta ou fuga” também confere um bom prognóstico,^20 , 21^ independentemente do uso de betabloqueadores.

A admissão aguda no serviço de emergência é uma situação de estresse onde se esperam respostas autonômicas que preparam o corpo para uma situação de luta ou fuga.^22^ Nesse contexto, aumentos na pressão de pulso e frequência cardíaca são esperados, aumentando a perfusão nos músculos esqueléticos e órgãos vitais.

Com base nas respostas hemodinâmicas fisiológicas inerentes a situações de luta ou fuga, propõe-se o cálculo do índice hemodinâmico agudo (IHA) a partir da frequência cardíaca e pressão de pulso. Nossa principal hipótese é que o IHA poderia representar um parâmetro prognóstico objetivo e intra-hospitalar a ser usado na descompensação aguda de pacientes com IC com fração de ejeção reduzida (ICFER). Portanto, o objetivo deste estudo foi avaliar a capacidade prognóstica intra-hospitalar do IHA na ICFER aguda descompensada.

## Métodos

Esta análise foi baseada no I Registro Brasileiro de Insuficiência Cardíaca (BREATHE),^23 , 24^ um estudo observacional transversal tipo registro da IC aguda com seguimento longitudinal que ocorreu de fevereiro de 2011 a dezembro de 2012. Para inclusão no registro, pacientes deveriam possuir mais de 18 anos e ter sido admitidos com IC descompensada; os pacientes não poderiam ter sido submetidos a cirurgia de revascularização miocárdica ou intervenção coronária percutânea no mês anterior, nem ter sido admitidos com um diagnóstico de sepse. Os critérios de Boston foram utilizados para a confirmação da IC.^25^ A participação no registro não requeria nenhum regime de tratamento especial. Os métodos detalhados, assim como os critérios de inclusão e exclusão, estão disponíveis na literatura.^24^ As informações sobre cada paciente foram incluídas num formulário de registro individual, disponível *online* .

Este estudo incluiu a análise da admissão hospitalar e do seguimento dos pacientes até a alta médica, morte ou transferência para outro hospital (o que foi verificado primeiro) dos pacientes com ICFER aguda descompensada. O desfecho primário do estudo foi a mortalidade intra-hospitalar.

**Figura 1:**
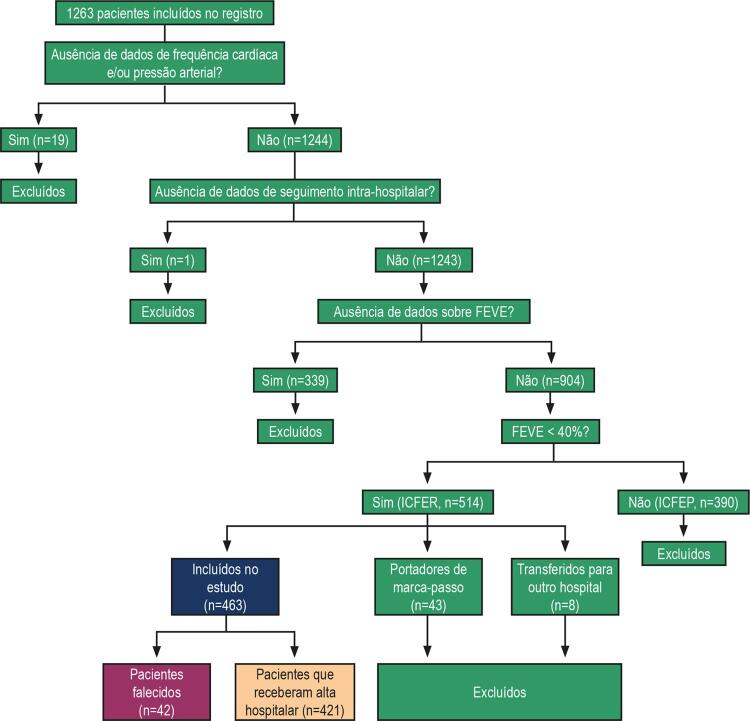
– Diagrama de seleção de pacientes para o registro. FEVE: fração de ejeção do ventrículo esquerdo; ICFER: insuficiência cardíaca com fração de ejeção reduzida; ICFEP: insuficiência cardíaca com fração de ejeção preservada.

Todos os pacientes do registro com evidência de fração de ejeção do ventrículo esquerdo menor que 40% foram incluídos nesta análise, exceto aqueles com informações faltantes (frequência cardíaca na admissão, pressão arterial, fração de ejeção ou interrupção do seguimento devido à transferência). Indivíduos com ritmo cardíaco controlado por marca-passo também foram excluídos, visto que não era esperado que suas frequências cardíacas possuíssem um controle autonômico ( Figura 1 ).

### Variáveis derivadas

Frequência cardíaca e pressão arterial sistólica e diastólica na admissão foram obtidas a partir da base de dados do registro e utilizadas no cálculo das variáveis derivadas da seguinte maneira: pressão de pulso = pressão arterial sistólica – pressão arterial diastólica; pressão de pulso proporcional = pressão de pulso / pressão arterial sistólica; IHA= (Pressão de pulso x frequência cardíaca) / 1000

### Aspectos éticos

Este estudo está de acordo com os princípios da Declaração de Helsinque. O estudo foi aprovado pelo Hospital do Coração, São Paulo (registro 144/2011) e pelo comitê de ética em pesquisa de cada instituição participante. Todos os pacientes assinaram um termo de consentimento esclarecido antes do recrutamento.

### Análise estatística

Inicialmente, utilizado o teste de Shapiro Wilk para verificar a normalidade da distribuição dos dados, validando o uso da estatística paramétrica. Variáveis contínuas foram descritas como médias e desvios-padrão, e variáveis categóricas foram descritas como proporções. Dados clínicos e demográficos dos pacientes que vieram a óbito durante a hospitalização (falecidos) e daqueles que receberam alta médica (vivos) foram comparados por um teste t de Student não-pareado ou qui-quadrado. Um valor-p bicaudal < 0,05 foi considerado estatisticamente significativo.

Depois de verificar a distribuição normal, os percentis 25º, 50º e 75º de frequência cardíaca e pressão arterial sistólica e diastólica foram utilizados na construção de curvas ROC ( *Receiver Operating Characteristic* ) usando a mortalidade intra-hospitalar como desfecho principal. O valor de corte do IHA foi definido pelo 50º percentil de sua distribuição. Sensibilidade, especificidade, e área sob a curva ROC (AUC) foram estabelecidas para cada valor de corte. Estatística c foi usada para comparar a capacidade prognóstica dos valores de corte de frequência cardíaca e pressão arterial com os valores de corte de IHA.

A análise de regressão foi realizada após a verificação de relação linear, normalidade multivariada, homoscedasticidade e da ausência de multicolinearidade e autocorrelação. Foram realizadas análises de regressão linear múltipla para testar a capacidade prognóstica independente de cada um dos valores de corte significativos de frequência cardíaca, pressão arterial e IHA. Esta análise também incluiu variáveis com significância estatística de acordo com testes t não-pareado e qui-quadrado. Visto que nem todos os pacientes apresentavam dados laboratoriais, estes não foram incluídos na análise de regressão. O critério de informação de Akaike (AIC)^26^ foi usado para comparar os modelos de regressão múltipla.

O *software* STATA versão 14.2 (StataCorp, Texas, EUA) foi utilizado em todas as análises estatísticas e gráficos.

## Resultados

O registro BREATHE incluiu 463 pacientes com ICFER admitidos em serviços de emergência no Brasil ( Tabela 1 ), com um índice de mortalidade intra-hospitalar de 9%. A principal causa de descompensação foi má aderência ao tratamento medicamentoso (em 37% dos pacientes que receberam alta e 31% daqueles que faleceram, p = 0,75). Outras causas importantes de descompensação foram infecções (em 21% dos pacientes que receberam alta e 24% daqueles que faleceram, p = 0,17) e ingestão excessiva de sal ou fluidos (em 11% dos pacientes que receberam alta e 12% daqueles que faleceram, p = 0,90).

**Tabela 1:** – Dados demográficos e clínicos dos pacientes com insuficiência cardíaca aguda descompensada com fração de ejeção reduzida

Variáveis	Total (N = 463)	Pacientes que receberam alta hospitalar (n = 421)	Pacientes falecidos (n = 42)	Valor-p
**Demográficas**				
Idade, anos ± DP	61 ± 16	61 ± 15	58 ± 17	0,27
Sexo masculino, n (%)	141 (30)	127 (30)	14 (33)	0,67
**Etiologia da insuficiência cardíaca**				
Isquêmica, n (%)	155 (33)	141 (33)	14 (33)	0,98
Doença de Chagas, n (%)	53 (11)	43 (10)	10 (24)	0,008
**Comorbidades**				
Hipertensão, n (%)	318 (69)	290 (69)	28 (67)	0,77
Fibrilação atrial, n (%)	109 (23)	101 (23)	8 (19)	0,51
Diabetes mellitus, n (%)	177 (38)	164 (39)	13 (31)	0,31
Insuficiência renal crônica, n (%)	98 (21)	81 (19)	17 (40)	0,001
Dislipidemia, n (%)	162 (35)	150 (36)	12 (29)	0,36
Depressão, n (%)	52 (11)	50 (12)	2 (5)	0,16
Histórico de acidente vascular cerebral, n (%)	56 (12)	46 (11)	10 (24)	0,015
Histórico de câncer, n (%)	18 (4)	14 (3)	4 (9)	0,048
**Tratamento**				
Betabloqueador, n (%)	273 (66)	241 (64)	32 (82)	0,023
Inibidor de ECA/BRA, n (%)	274 (59)	251 (60)	23 (55)	0,50
Diuréticos tiazídicos e de alça, n (%)	311 (67)	277 (66)	34 (81)	0,046
Bloqueadores de canais de cálcio, n (%)	28 (7)	25 (7)	3 (8)	0,80
Digitálicos, n (%)	121 (29)	102 (27)	19 (50)	0,005
Espironolactona, n(%)	182 (44)	156 (41)	26 (67)	0,002
Estatinas, n (%)	139 (33)	127 (34)	12 (31)	0,71
**Hemodinâmica**				
Frequência cardíaca, bpm ± DP	90 ± 23	90 ± 23	82 ± 21	0,025
Pressão arterial sistólica, mmHg ± DP	121 ± 29	122 ± 30	112 ± 26	0,036
Pressão arterial diastólica, mmHg ± DP	76 ± 19	77 ± 19	70 ± 14	0,020
Pressão de pulso, mmHg ± DP	45 ± 18	45 ± 18	43 ± 18	0,30
Pressão de pulso proporcional, % ± DP	37 ± 9	37 ± 9	37 ± 8	0,75
IHA, mmHg⋅bpm ± DP	4 ± 2	4 ± 2	3 ± 2	0,08
IHA < 4 mmHg⋅bpm, n (%)	273 (60)	240 (57)	33 (79)	0,007
FEVE, % ± DP	27 ± 8	27 ± 8	25 ± 6	0,20
**Perfil hemodinâmico**				
A, %	49 (11)	45 (11)	4 (10)	0,81
B, %	311 (67)	288 (68)	23 (55)	0,07
C, %	81 (17)	68 (16)	13 (30)	0,02
L, %	22 (5)	20 (5)	2 (5)	0,99
Exames laboratoriais*				
Hematócrito, % ± DP	40 ± 7	40 ± 6	38 ± 9	0,07
Hemoglobina, g/dL ± DP	13 ± 2	13 ± 2	13 ± 2	0,26
Creatinina, mg/dL ± DP	1,5 ± 0,9	1,5 ± 0,8	1,9 ± 0,9	0,001
Ureia, mg/dL ± DP	68 ± 41	65 ± 38	100 ± 50	<0,001
Sódio, mEq/L ± DP	137 ± 13	138 ± 14	136 ± 6	0,51

DP: desvio padrão; ECA: enzima conversora da angiotensina; BRA: bloqueadores dos receptores da angiotensina; IHA: índice hemodinâmico agudo; FEVE: fração de ejeção do ventrículo esquerdo. Valor-p calculado a partir da comparação univariada entre ambos os grupos.*N=412.

Os pacientes falecidos apresentavam mais comorbidades, assim como frequência cardíaca e pressão arterial sistólica e diastólica mais altas que aqueles que sobreviveram. Considerando o 50º percentil do IHA como valor de corte (4 mmHg⋅bpm), quase 80% dos pacientes falecidos possuíam um baixo IHA.

**Figura 2:**
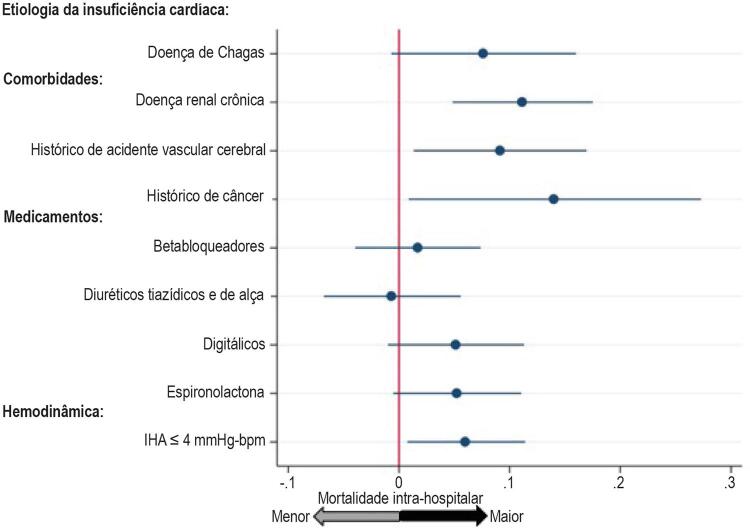
– Razão de chance, calculada a partir de um modelo de regressão multivariada que incluiu etiologia da insuficiência cardíaca, comorbidades, uso de medicamentos e índice hemodinâmico de pacientes admitidos com insuficiência cardíaca aguda descompensada com fração de ejeção reduzida (N = 463). IHA: índice hemodinâmico agudo.

**Figura 3:**
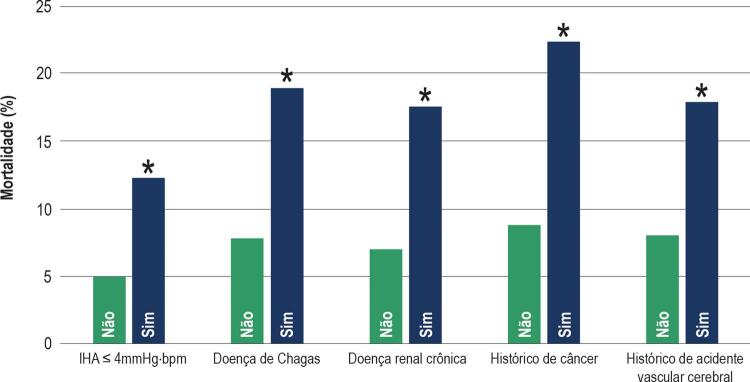
– Índices de mortalidade intra-hospitalar em pacientes com insuficiência cardíaca aguda descompensada com fração de ejeção reduzida de acordo com a presença de fatores prognósticos. *p < 0,05 quando comparado ao “Não” no mesmo índice prognóstico.

Visto que o cálculo do IHA inclui valores de frequência cardíaca e pressão arterial, foram comparadas as AUC de IHA ≤ 4 mmHg⋅bpm e de diferentes valores de corte de frequência cardíaca e pressão arterial sistólica e diastólica ( Tabela 2 ). IHA ≤ 4 mmHg⋅bpm foi um melhor preditor de mortalidade intra-hospitalar que frequência cardíaca ≤ 88 bpm, mas teve desempenho semelhante a outros valores de corte de pressão arterial. Quando estes fatores prognósticos hemodinâmicos foram incluídos na análise multivariada, apenas o IHA manteve capacidade prognóstica independente ( Tabela 3 ). O modelo de regressão que incluiu a etiologia de doença de Chagas, comorbidades, uso de medicamentos e IHA mostrou uma melhor capacidade prognóstica de mortalidade intra-hospitalar que os outros modelos propostos (Modelo 0: sem IHA; Modelos 1-4: com parâmetros hemodinâmicos adicionados ao Modelo 0). Doença renal crônica, histórico de câncer ou acidente vascular cerebral permaneceram como preditores independentes de mortalidade intra-hospitalar em todos os modelos propostos. IHA < 4 mmHg⋅bpm esteve independentemente relacionado à mortalidade intra-hospitalar neste registro mesmo após o ajuste para a etiologia da IC, comorbidades e uso de medicamentos ( Figura 2 ). Pacientes admitidos com baixos IHAs tinham probabilidade de óbito de 12,1%, um valor 250% maior que pacientes admitidos com IHAs maiores que 4 mmHg⋅bpm (4,8%, p = 0,008, Figura 3 ). Como trata-se de um estudo tipo registro, não houve intervenções no tratamento recebido pelos pacientes. Medicamentos inotrópicos foram utilizados em 11% dos pacientes que receberam alta e em 28% dos que vieram a óbito (p < 0,001).

**Tabela 2:** – Sensibilidade, especificidade, AUC com IC de 95% e melhores valores de corte para mortalidade intra-hospitalar em pacientes com insuficiência cardíaca aguda descompensada com fração de ejeção reduzida

Parâmetros prognósticos propostos	Análise univariada				Comparação com AUC considerando valor de IHA ≤ 4 mmHg⋅bpm
	Sensibilidade	Especificidade	AUC (IC 95%)	Valor-p	Valor-p
IHA ≤ 4 mmHg⋅bpm	0,786	0,429	0,607 (0,540-0,674)	0,01	----
**Frequência cardíaca**					
≤ 74 bpm	0,309	0,750	0,530 (0,456-0,604)	0,39	----
≤ 88 bpm	0,667	0,513	0,590 (0,514-0,666)	0,03	0,048
≤ 104 bpm	0,857	0,254	0,556 (0,498-0,613)	0,58	----
**Pressão arterial sistólica**					
≤ 100	0,452	0,698	0,575 (0,496-0,654)	0,04	0,450
≤ 120	0,738	0,430	0,584 (0,513-0,655)	0,04	0,570
≤ 140	0,905	0,190	0,547 (0,498-0,596)	0,14	----
**Pressão arterial diastólica**					
≤ 60	0,453	0,741	0,596 (0,518-0,676)	0,01	0,830
≤ 73	0,643	0,513	0,578 (0,500-0,655)	0,06	----
≤ 84	0,857	0,257	0,557 (0,499-0,614)	0,11	----

*AUC: área sob a curva ROC (Receiving Operating Characteristic); IC: intervalo de confiança; IHA: índice hemodinâmico agudo.*

**Tabela 3:** – Análise multivariada para predição da mortalidade intra-hospitalar incluindo diferentes parâmetros hemodinâmicos não-invasivos.

	Modelo 1		Modelo 2		Modelo 3		Modelo 4		Modelo 5	
AIC	137,0		136,3		135,6		135,7		133,7	
Valor-p em comparação com Modelo 0	0,294		0,183		0,113		0,116		0,035	
Parâmetro	RC IC 95%	p	RC IC 95%	p	RC IC 95%	p	RC IC 95%	p	RC IC 95%	p
Doença de Chagas	0,089 0,006-0,171	0,035	0,784 -0,006-0,163	0,071	0,080 -0,003-0,164	0,060	0,777 -0,006-0,162	0,071	0,765 -0,007-0,160	0,072
DRC	0,104 0,041-0,167	0,001	0,104 0,040-0,167	0,001	0,107 0,044-0,170	0,001	0,100 0,037-0,164	0,002	0,112 0,048-0,175	0,001
Histórico de acidente vascular cerebral	0,840 0,054-0,163	0,036	0,089 0,011-0,168	0,025	0,093 0,014-0,170	0,021	0,858 0,007-0,164	0,032	0,092 0,013-0,169	0,022
Histórico de câncer	0,143 0,011-0,276	0,033	0,148 0,016-0,281	0,028	0,139 0,007-0,271	0,039	0,139 0,007-0,272	0,038	0,140 0,009-0,273	0,037
Betabloqueadores	0,168 -0,40-0,073	0,563	0,196 -0,037-0,076	0,497	0,180 -0,038-0,074	0,531	0,021 -0,035-0,077	0,463	0,172 -0,394-0,073	0,551
Diuréticos tiazídicos e de alça	-0,005 -0,066-0,057	0,887	-0,003 -0,065-0,058	0,918	-0,005 -0,066-0,057	0,884	-0,004 -0,065-0,058	0,909	-0,006 -0,068-0,056	0,850
Digitálicos	0,053 -0,009-0,115	0,096	0,056 -0,005-0,117	0,073	0,538 -0,007-0,115	0,086	-0,003 -0,007-0,115	0,086	0,515 -0,009-0,113	0,100
Espironolactona	0,540 -0,004-0,112	0,068	0,527 -0,005-0,110	0,075	0,053 -0,004-0,111	0,071	0,053 -0,004-0,111	0,072	0,053 -0,005-0,110	0,074
Frequência cardíaca ≤ 88 bpm	0,277 -0,025-0,080	0,627								
Pressão arterial sistólica ≤ 100 mmHg			0,380 -0,018-0,094	0,188						
≤ 120 mmHg					0,042 -0,010-0,095	0,117				
Pressão arterial diastólica ≤ 60 mmHg							0,046 -0,012-0,105	0,121		
IHA ≤ 4 mmHg⋅bpm									0,061 0,007-0,114	0,025

*AIC: critério de informação de Akaike; RC: razão de chance; IC: intervalo de confiança; DRC: doença renal crônica; IHA: índice hemodinâmico agudo. O Modelo 0 incluiu doença de Chagas como etiologia da insuficiência cardíaca, doença renal crônica, histórico de câncer, uso contínuo de betabloqueadores, diuréticos tiazídicos e de alça, digitálicos e espironolactona. Os Modelos 1 a 5 incluíram todas as variáveis do Modelo 0 mais um parâmetro e valor de corte da seguinte forma: o Modelo 1 considerou frequência cardíaca ≤ 88 bpm; o Modelo 2 considerou pressão arterial sistólica ≤ 100 mmHg, o Modelo 3 considerou pressão arterial sistólica ≤ 120 mmHg, o Modelo 4 considerou pressão arterial diastólica ≤ 60 mmHg e o Modelo 5 considerou IHA ≤ 4 mmHg⋅bpm.*

## Discussão

Este estudo apresentou o IHA e demonstrou que este é um preditor independente de mortalidade intra-hospitalar em pacientes com ICFER aguda descompensada. A mortalidade intra-hospitalar de pacientes com IC aguda descompensada é elevada, como demonstrado pelo registro BREATHE e por estudos realizados em outros países.^27^ Diferentes razões contribuem para uma alta mortalidade a curto prazo de pacientes com IC aguda descompensada, como idade, comorbidades e tempo entre início dos sintomas e admissão hospitalar.^27^ Visto que o manejo de pacientes com IC aguda pode incluir procedimentos invasivos e de alto custo, como o suporte circulatório mecânico, a validação de índices prognósticos que possam auxiliar em decisões terapêuticas é de extrema importância.^28^

A IC aguda descompensada pode ser manejada por especialistas em IC, cardiologistas, intensivistas, médicos emergencistas ou clínicos; em serviços de emergência, enfermarias ou unidades de tratamento intensivo.^2^ O treinamento destes profissionais e a disponibilidade de recursos nestes ambientes pode variar substancialmente. Quando somados à diversidade de casos de pacientes, estes aspectos tornam difícil a criação de escores prognósticos que possam ser utilizados amplamente. Por exemplo, apesar do recente interesse no uso de biomarcadores,^29^ a avaliação destes pode não estar disponível em estabelecimentos de saúde afastados dos grandes centros ou com poucos recursos. Nohria et al.^6^ apresentaram uma abordagem clínica prática para classificar os pacientes em perfis hemodinâmicos, permitindo a predição do prognóstico e guiando o tratamento da IC aguda. Esta abordagem depende da experiência do médico responsável^8 , 9^ e pode perder sua utilidade quando se trata de um médico não-especialista em IC. Nossos resultados corroboram a falta de acurácia do exame físico cardiovascular,^9^ visto que 11% dos pacientes foram classificados como de perfil hemodinâmico A e ainda assim considerados casos de IC aguda descompensada.

A medição da frequência cardíaca e a pressão arterial está disponível em praticamente qualquer estabelecimento de saúde, tem boa acurácia e requer treinamento mínimo.^11 , 12^ Estudos anteriores tentaram utilizar estes parâmetros como fatores prognósticos na IC aguda descompensada. A relação entre frequência cardíaca e prognóstico é conhecida há décadas, e com o advento dos betabloqueadores e, mais recentemente, da ivabradina, frequências cardíacas baixas são um alvo no tratamento da IC estável.^19^ Além disso, a incompetência cronotrópica também é um marcador de risco: pacientes cujas frequências cardíacas não aumentam durante o exercício físico têm prognósticos piores que aqueles com frequências cardíacas de reserva normais, mesmo com o uso de betabloqueadores.^20 , 21^ Apesar de estudos terem determinado o aumento esperado na frequência cardíaca durante um teste de esforço,^20 , 21^ não há valores de referência para o aumento da frequência cardíaca em situações do tipo “luta ou fuga” como é o caso das admissões em serviços de emergência. Pacientes japoneses com IC aguda descompensada admitidos com frequências cardíacas acima de 120 bpm apresentaram menores índices de mortalidade que aqueles admitidos com frequências cardíacas menores.^15^ Entretanto, uma alta frequência cardíaca foi considerada um preditor independente de mortalidade a curto prazo em pacientes com IC aguda descompensada em outros estudos.^16 , 30 , 31^

O registro OPTIMIZE-HF^14^ observou que pressão arterial sistólica abaixo de 120 mmHg identificava pacientes com IC aguda descompensada que possuíam um prognóstico ruim apesar dos tratamentos. Baixa pressão arterial sistólica também indicou risco a curto prazo numa coorte europeia.^13^ Neste estudo, a pressão arterial abaixo de 120 mmHg não estava independentemente relacionada à mortalidade de acordo com a análise multivariada. Pacientes do registro BREATHE eram mais jovens, e os protocolos de tratamento estavam mais atualizados que aqueles utilizados nos outros estudos (quase uma década antes). Além disso, esses dois estudos^13 , 14^ incluíram pacientes com fração de ejeção preservada ou reduzida, e o valor prognóstico da pressão arterial pode variar de acordo com a fração de ejeção do ventrículo esquerdo.^32^ Baixa pressão de pulso foi definida como um preditor independente de mortalidade na IC aguda descompensada pelo estudo VMAC-HF.^33^ O tratamento da IC evoluiu substancialmente desde então, o que pode explicar a falta de poder prognóstico da pressão de pulso em nosso estudo.

A interação intrínseca entre pressão arterial e frequência cardíaca e como elas são influenciadas por medicamentos para IC pode ter influenciado os resultados de estudos prévios considerando cada um destes parâmetros. Até onde sabemos, este é o primeiro estudo a apresentar um índice que combina a análise da frequência cardíaca e pressão de pulso de pacientes com IC aguda descompensada e demonstrar que sua capacidade como ferramenta prognóstica é superior àquelas da frequência cardíaca ou pressão arterial sozinhas.

### Limitações

Esta análise possui limitações. Primeiramente, a mortalidade intra-hospitalar foi baseada no registro feito por pesquisadores e não foi confirmada. Na realidade, registros são estudos observacionais, e análises quanto à adequação do tratamento a cada paciente não estavam no escopo de nosso estudo. Considerando que nosso principal objetivo foi analisar a utilidade de um índice facilmente obtido no momento da admissão do paciente no setor de emergência e que os testes de troponina e BNP (peptídeo natriurético cerebral) não estão disponíveis em todos os estabelecimentos de saúde brasileiros, parâmetros laboratoriais não foram incluídos em nosso modelo.

Os dados do registro não foram obtidos através de um protocolo padronizado, portanto as medições de pressão arterial e frequência cardíaca podem ter sido realizadas com equipamentos diferentes. De qualquer maneira, estes são sinais vitais que requerem treinamento mínimo para sua medição.^11 , 12^ Além disso, o fato de não existir uma padronização neste caso aumenta a aplicabilidade clínica do nosso índice, já que reflete resultados reais.

É importante notar que os resultados apresentados neste estudo se restringem a pacientes com fração de ejeção reduzida. O estudo foi conduzido entre 2011 e 2012, antes da aprovação de medicamentos novos como a ivabradina e sacubitril-valsartana,^19^ que poderiam influenciar o IHA.

A população brasileira é muito diversa quanto à etnia e acesso a estabelecimentos de saúde, e este estudo incluiu hospitais públicos e privados de todas as regiões do país.^23^ Embora a generalização de nossos resultados possa ser limitada, é importante enfatizar que os dados clínicos e demográficos dos pacientes incluídos neste estudo são semelhantes aos de outras coortes.^14 , 16 , 30 , 31^

É importante ressaltar que a AUC da análise do IHA foi relativamente pequena. Mesmo assim, sua sensibilidade foi boa e pode ser útil na orientação de médicos emergencistas na triagem de pacientes.

## Conclusão

Diferentes fatores prognósticos têm sido propostos para a IC aguda descompensada. Entretanto, muitos dependem de biomarcadores, treinamento de pessoal e recursos tecnológicos, que nem sempre estão disponíveis. O IHA consiste num fator prognóstico de mortalidade intra-hospitalar prático, objetivo e facilmente obtido em pacientes com IC aguda descompensada. Futuros estudos prospectivos deverão avaliar a reprodutibilidade destes resultados em outras populações.
