# Supplementary material for: Índice Hemodinâmico Agudo Prediz Mortalidade Intra-Hospitalar de Pacientes com Insuficiência Cardíaca Aguda Descompensada
Source: Arq Bras Cardiol. 2021 Jan 27;116(1):77–86. [Article in Portuguese] doi: 10.36660/abc.20190439 (PMC8159496; doi:10.36660/abc.20190439)
Supplement: Translation

# Translation: Acute Hemodynamic Index Predicts In-Hospital Mortality in Acute Decompensated Heart Failure

**Authors:** Renata R. T. Castro, Luka Lechnewski, Alan Homero, Denilson Campos de Albuquerque, Luis Eduardo Rohde, Dirceu Almeida, João David, Salvador Rassi, Fernando Bacal, Edimar Bocchi, Lidia Moura

**Affiliations:** 1 USA Brigham and Womens Hospital – Medicine, Boston - USA; 2 Brazil Hospital Naval Marcilio Dias, Rio de Janeiro, RJ - Brazil; 3 Brazil Faculdade de Medicina, Universidade Iguaçu, Nova Iguaçu, RJ - Brazil; 4 Brazil Pontifícia Universidade Católica do Paraná, Curitiba, PR - Brazil; 5 Brazil Universidade Estadual do Rio de Janeiro, Rio de Janeiro, RJ - Brazil; 6 Brazil Universidade Federal do Rio Grande do Sul, Porto Alegre, RS - Brazil; 7 Brazil Universidade Federal de São Paulo, São Paulo, SP – Brazil; 8 Brazil Hospital de Messejana, Fortaleza, CE - Brazil; 9 Brazil Universidade Federal de Goiás, Goiânia, GO - Brazil; 10 Brazil Universidade de São Paulo Instituto do Coração, São Paulo, SP – Brazil

**Keywords:** Heart Failure, Heart Rate, Blood Pressure, Prognosis, Mortality

## Abstract

**Background:**

The physical examination enables prognostic evaluation of patients with decompensated heart failure (HF), but lacks reliability and relies on the professional’s clinical experience. Considering hemodynamic responses to “fight or flight” situations, such as the moment of admission to the emergency room, we proposed the calculation of the acute hemodynamic index (AHI) from values of heart rate and pulse pressure.

**Objective:**

To evaluate the in-hospital prognostic ability of AHI in decompensated HF.

**Methods:**

A prospective, multicenter, registry-based observational study including data from the BREATHE registry, with information from public and private hospitals in Brazil. The prognostic ability of the AHI was tested by receiver-operating characteristic (ROC) analyses, C-statistics, Akaike’s information criteria, and multivariate regression analyses. p-values < 0.05 were considered statistically significant.

**Results:**

We analyzed data from 463 patients with heart failure with low ejection fraction. In-hospital mortality was 9%. The median AHI value was used as cut-off (4 mmHg⋅bpm). A low AHI (≤ 4 mmHg⋅bpm) was found in 80% of deceased patients. The risk of in-hospital mortality in patients with low AHI was 2.5 times that in patients with AHI > 4 mmHg⋅bpm. AHI independently predicted in-hospital mortality in acute decompensated HF (sensitivity: 0.786; specificity: 0.429; AUC: 0.607 [0.540–0.674]; p = 0.010) even after adjusting for comorbidities and medication use [OR: 0.061 (0.007–0.114); p = 0.025).

**Conclusions:**

The AHI independently predicts in-hospital mortality in acute decompensated HF. This simple bed-side index could be useful in an emergency setting. (Arq Bras Cardiol. 2021; 116(1):77-86)

## Introduction

Heart failure (HF) is one of the main reasons for emergency admissions in the Western world.^1^Although previous studies have shown that treatment by a HF specialist can lead to better results, most cases of acute decompensated HF are originally evaluated and managed by emergency physicians^2 , 3^in facilities with different levels of resource availability.

Despite recent advances in technology and medical devices, the physical examination remains the cornerstone of the evaluation of patients with HF.^4 , 5^Physicians evaluate congestion and perfusion from the patient’s history and a physical examination, assigning hemodynamic profiles that guide therapy and provide prognostic information in an acute HF setting.^6^Although practical, the physician’s assessment of perfusion lacks reliability^7^and depends on clinician experience,^8 , 9^providing subjective information.^10^Therefore, objective prognostic parameters that can be easily obtained in the emergency room would be useful in the management of acute HF.

Blood pressure and heart rate are parameters that can be easily obtained by any healthcare professional with good reproducibility and accuracy.^11 , 12^Systolic blood pressure is an independent predictor of in-hospital and post-discharge outcomes in acute heart failure.^13 , 14^Additionally, low blood pressure and narrow proportional pulse pressure are markers of low perfusion.^4 , 6 , 9^

The relationship between admission resting heart rate and the prognosis of patients with HF is not as straightforward. In fact, the literature is controversial, showing that a high admission heart rate can be related to worse or better prognoses.^15 - 17^Although low resting heart rates reduce risk in patients with stable chronic HF with reduced ejection fraction (HFrEF),^18 , 19^the ability to increase heart rate during a “fight or flight” reaction certainly confers good prognosis,^20 , 21^regardless of the use of beta-blockers.

Acute admission to the emergency room is a stressful situation, expected to elicit autonomic responses that prepare the body to fight or flight.^22^Increases in pulse pressure and heart rate are thus expected in this scenario, augmenting perfusion in skeletal muscles and vital organs.

Based on the physiological hemodynamic responses inherent to “fight or flight” situations, we have proposed the calculation of the acute hemodynamic index (AHI) using heart rate and pulse pressure. Our main hypothesis was that AHI could be an objective in-hospital prognostic parameter to be used in patients with acute decompensated HFrEF. Therefore, we aimed to evaluate the in-hospital prognostic ability of AHI in acute decompensated HFrEF.

## Methods

This analysis is based on the I Brazilian registry of HF (BREATHE Registry),^23 , 24^a cross-sectional, observational acute HF registry with longitudinal follow-up that happened from February 2011 to December 2012. For inclusion in the registry, patients should be over 18 years old and have been admitted with decompensated HF; patients should not have been submitted to a coronary artery bypass graft of percutaneous coronary intervention in the previous month or have been admitted with a sepsis diagnosis. Boston criteria were used for HF confirmation.^25^Participation in the registry did not require any special treatment regimen. Detailed methods, as well as exclusion and inclusion criteria, have been previously described.^24^Data on each patient are available online in individual registration forms.

This study includes the analysis of patients with acute decompensated HFrEF from hospital admission and follow-up until discharge, death, or transfer to another hospital (whichever happened first). The primary endpoint of the study was in-hospital mortality.

All patients in the registry with evidence of left ventricle ejection fraction < 40% were included in the present analysis, except those with missing information (admission heart rate, blood pressure, ejection fraction, or loss of follow-up due to transfer to another hospital). Individuals with pacemaker-controlled heart rhythm were also excluded, as their heart rate was not expected to be autonomic-driven ( Figure 1 ).

**Figure 1:** – Patient selection flowchart. LVEF: left ventricular ejection fraction; HFrEF: heart failure with reduced ejection fraction; HFpEF: heart failure with preserved ejection fraction.

### Derived variables

Heart rate and systolic and diastolic blood pressure at admission were available from the registry database and were used for calculating derived variables as follows: pulse pressure = systolic blood presssure – diastolic blood pressure; proportional pulse pressure = pulse pressure / systolic blood pressure; AHI= (pulse pressure x heart rate) / 1000.

### Ethics

This investigation conforms to the principles outlined in the Declaration of Helsinki. The study was approved by the Hospital do Coração, São Paulo (registry 144/2011) and the Institutional Review Board of each participating institution. All patients signed an informed consent form before enrollment.

### Statistical analyses

Initially, a Shapiro-Wilk test was used to verify the normality of data distribution and validate the use of parametric statistics. Continuous variables were reported as means and standard deviations, while categorical variables were reported as proportions. Clinical and demographic data from patients who died during the hospitalization period (deceased) and those who were successfully discharged (alive) were compared using unpaired Student’s t-tests or chi-squared tests. A two-sided p-value < 0.05 was considered significant.

After verifying a normal distribution, the 25th, 50th, and 75th percentiles of heart rate and systolic and diastolic blood pressure were used to construct receiver-operating characteristic (ROC) curves using in-hospital mortality as the main outcome. The cut-off value defined for the AHI was its 50th percentile. Sensitivity, specificity, and area under the ROC curve (AUC) were reported for each cut-off value. C-statistics were used to compare the prognostic ability of heart rate and blood pressure cut-off values to the AHI cut-off values.

Regression analyses were performed after verifying for linear relationships, multivariate normality, homoscedasticity, and the absence of multicollinearity and autocorrelation.

Multiple linear regression analyses were performed to test the independent prognostic ability of each significant cut-off value regarding heart rate, blood pressure, and AHI. This analysis included variables with statistical significance according to the previously cited unpaired Student’s t-tests or chi-squared tests. As laboratory results were not available for all patients, they were not included in the regression analysis. Akaike’s information criterion (AIC)^26^was used to compare multiple regression models. All statistical analyses and graphs were performed using STATA 14.2 (StataCorp, Texas, USA).

## Results

The BREATHE registry included 463 patients with HFrEF admitted to emergency services in Brazil ( Table 1 ), with an in-hospital mortality index of 9%. The main reason for decompensation was poor medication adherence (37% of discharged patients vs 31% of deceased patients, p = 0.75). Other important causes of decompensation were infection (21% of discharged patients vs 24% of decease patients, p = 0.17) and excessive salt or fluid intake (11% of discharhed patients vs 12% of deceased patients, p = 0.9).

**Table 1:** – Demographic and clinical data of patients with acute decompensated heart failure with reduced ejection fraction

Characteristics	All patients (n = 463)	Discharged patients (n = 421)	Deceased patients (n = 42)	p-value
**Demographic**				
Age, years ± SD	61 ± 16	61 ± 15	58 ± 17	0.27
Male sex, n (%)	141 (30)	127 (30)	14 (33)	0.67
**Heart failure etiology**				
Ischemic, n (%)	155 (33)	141 (33)	14 (33)	0.98
Chagas disease, n (%)	53 (11)	43 (10)	10 (24)	0.008
**Comorbidities**				
Hypertension, n (%)	318 (69)	290 (69)	28 (67)	0.77
Atrial fibrillation, n (%)	109 (23)	101 (23)	8 (19)	0.51
Diabetes mellitus, n (%)	177 (38)	164 (39)	13 (31)	0.31
Chronic kidney failure, n (%)	98 (21)	81 (19)	17 (40)	0.001
Dyslipidemia, n (%)	162 (35)	150 (36)	12 (29)	0.36
Depression, n (%)	52 (11)	50 (12)	2 (5)	0.16
History of stroke, n (%)	56 (12)	46 (11)	10 (24)	0.015
History of cancer, n (%)	18 (4)	14 (3)	4 (9)	0.048
**Treatment**				
Beta-blocker, n (%)	273 (66)	241 (64)	32 (82)	0.023
ACEi/ARB, n (%)	274 (59)	251 (60)	23 (55)	0.50
Loop/thiazide diuretics, n (%)	311 (67)	277 (66)	34 (81)	0.046
Calcium channel blockers, n (%)	28 (7)	25 (7)	3 (8)	0.80
Digitalis, n (%)	121 (29)	102 (27)	19 (50)	0.005
Spironolactone, n (%)	182 (44)	156 (41)	26 (67)	0.002
Statins, n (%)	139 (33)	127 (34)	12 (31)	0.71
**Hemodynamics**				
Heart rate, bpm ± SD	90 ± 23	90 ± 23	82 ± 21	0.025
Systolic blood pressure, mmHg ± SD	121 ± 29	122 ± 30	112 ± 26	0.036
Diastolic blood pressure, mmHg ± SD	76 ± 19	77 ± 19	70 ± 14	0.020
Pulse pressure, mmHg ± SD	45 ± 18	45 ± 18	43 ± 18	0.30
Proportional pulse pressure, % ± SD	37 ± 9	37 ± 9	37 ± 8	0.75
AHI, mmHg⋅bpm ± SD	4 ± 2	4 ± 2	3 ± 2	0.08
AHI < 4 mmHg⋅bpm, n (%)	273 (60)	240 (57)	33 (79)	0.007
LVEF, % ± SD	27 ± 8	27 ± 8	25 ± 6	0.20
**Hemodynamic profile**				
A, %	49 (11)	45 (11)	4 (10)	0.81
B, %	311 (67)	288 (68)	23 (55)	0.07
C, %	81 (17)	68 (16)	13 (30)	0.02
L, %	22 (5)	20 (5)	2 (5)	0.99
Laboratory results*				
Hematocrit, % ± SD	40 ± 7	40 ± 6	38 ± 9	0.07
Hemoglobin, g/dL ± SD	13 ± 2	13 ± 2	13 ± 2	0.26
Creatinine, mg/dL ± SD	1.5 ± 0.9	1.5 ± 0.8	1.9 ± 0.9	0.001
Urea, mg/dL ± SD	68 ± 41	65 ± 38	100 ± 50	<0.001
Sodium, mEq/L ± SD	137 ± 13	138 ± 14	136 ± 6	0.51

ACEi: angiotensin converting enzyme inhibitors; ARB: angiotensin II receptor blockers; AHI: acute hemodynamic index; LVEF: left ventricular ejection fraction; SD: standard deviation. p-values were obtained in the univariate comparison between both groups. *N = 412.

Deceased patients presented more comorbidities and higher values of heart rate and systolic and diastolic blood pressure when compared to survivors. Considering the AHI’s 50th percentile, its cut-off value was 4 mmHg⋅bpm; almost 80% of the deceased patients had a low AHI.

As the AHI calculation included heart rate and blood pressure values, we compared the AUC of AHI ≤ 4 mmHg⋅bpm as a cut-off value with the AUC of different cut-off values of heart rate and systolic and diastolic blood pressure ( Table 2 ). AHI ≤ 4 mmHg⋅bpm was a better predictor of in-hospital mortality than heart rate ≤ 88 bpm, but had similar results when compared to prognostic cut-off values of blood pressure. When these hemodynamic prognostic factors were included in multivariate analyses, only AHI kept an independent prognostic ability ( Table 3 ). The regression model including Chagas disease etiology, comorbidities, medications, and AHI showed a better predictive capacity for in-hospital mortality than the other proposed models (Model 0: without AHI; Models 1–4: with hemodynamic parameters added to model 0). Chronic kidney disease and a history of cancer or stroke remained as independent in-hospital mortality predictors in all proposed models. AHI ≤ 4 mmHg⋅bpm was independently related to in-hospital mortality in this registry even after adjusting for HF etiology, comorbidities, and medication use ( Figure 2 ). Patients admitted with low AHI had a 12.1% chance of dying, which was 250% higher than that for patients with AHI > 4 mmHg⋅bpm (4.8%, p = 0.008, Figure 3 ). As this was a registry study, the research protocol did not intervene in the treatment received by patients. Inotropes were used in 11% of discharged patients and 28% of deceased patients (p < 0.001).

**Table 2:** – Sensitivity, specificity, AUC with 95% CI, and best cut-off values for in-hospital mortality in patients with acute decompensated heart failure with reduced ejection fraction

Proposed prognostic parameters	Univariate analysis				Comparison to AUC for AHI ≤ 4 mmHg⋅bpm
	Sensitivity	Specificity	AUC (95% CI)	p-value	p-value
AHI ≤ 4 mmHg⋅bpm	0.786	0.429	0.607 (0.540–0.674)	0.01	----
**Heart rate**					
≤ 74 bpm	0.309	0.750	0.530 (0.456–0.604)	0.39	----
≤ 88 bpm	0.667	0.513	0.590 (0.514–0.666)	0.03	0.048
≤ 104 bpm	0.857	0.254	0.556 (0.498–0.613)	0.58	----
**Systolic blood pressure**					
≤ 100	0.452	0.698	0.575 (0.496–0.654)	0.04	0.450
≤ 120	0.738	0.430	0.584 (0.513–0.655)	0.04	0.570
≤ 140	0.905	0.190	0.547 (0.498–0.596)	0.14	----
**Diastolic blood pressure**					
≤ 60	0.453	0.741	0.596 (0.518–0.676)	0.01	0.830
≤ 73	0.643	0.513	0.578 (0.500–0.655)	0.06	----
≤ 84	0.857	0.257	0.557 (0.499–0.614)	0.11	----

*AUC: area under receiver-operating characteristic curves; CI: confidence interval; AHI: acute hemodynamic index.*

**Table 3:** – Multivariate models for in-hospital mortality prediction including different non-invasive hemodynamic parameters

	Model 1		Model 2		Model 3		Model 4		Model 5	
AIC	137.0		136.3		135.6		135.7		133.7	
p-value vs Model 0	0.294		0.183		0.113		0.116		0.035	
Parameter	OR 95% CI	p	OR 95% CI	p	OR 95% CI	p	OR 95% CI	p	OR 95% CI	p
Chagas disease	0.089 0.006–0.171	0.035	0.784 -0.006–0.163	0.071	0.080 -0.003–0.164	0.060	0.777 -0.006–0.162	0.071	0.765 -0.007–0.160	0.072
CKD	0.104 0.041–0.167	0.001	0.104 0.040–0.167	0.001	0.107 0.044–0.170	0.001	0.100 0.037–0.164	0.002	0.112 0.048–0.175	0.001
History of stroke	0.840 0.054–0.163	0.036	0.089 0.011–0.168	0.025	0.093 0.014–0.170	0.021	0.858 0.007–0.164	0.032	0.092 0.013–0.169	0.022
History of cancer	0.143 0.011–0.276	0.033	0.148 0.016–0.281	0.028	0.139 0.007–0.271	0.039	0.139 0.007–0.272	0.038	0.140 0.009–0.273	0.037
Beta-blockers	0.168 -0.40–0.073	0.563	0.196 -0.037–0.076	0.497	0.180 -0.038–0.074	0.531	0.021 -0.035–0.077	0.463	0.172 -0.394–0.073	0.551
Loop and thiazide diuretics	-0.005 -0.066–0.057	0.887	-0.003 -0.065–0.058	0.918	-0.005 -0.066–0.057	0.884	-0.004 -0.065–0.058	0.909	-0.006 -0.068–0.056	0.850
Digitalis	0.053 -0.009–0.115	0.096	0.056 -0.005–0.117	0.073	0.538 -0.007–0.115	0.086	-0.003 -0.007–0.115	0.086	0.515 -0.009–0.113	0.100
Spironolactone	0.540 -0.004–0.112	0.068	0.527 -0.005–0.110	0.075	0.053 -0.004–0.111	0.071	0.053 -0.004–0.111	0.072	0.053 -0.005–0.110	0.074
Heart rate ≤ 88 bpm	0.277 -0.025–0.080	0.627								
Systolic blood pressure ≤ 100 mmHg			0.380 -0.018–0.094	0.188						
≤ 120 mmHg					0.042 -0.010–0.095	0.117				
Diastolic blood pressure ≤ 60 mmHg							0.046 -0.012–0.105	0.121		
AHI ≤ 4 mmHg*bpm									0.061 0.007–0.114	0.025

*AIC: Akaike’s information criterion; OR: odds ratio; CI: confidence interval; CKD: chronic kidney disease; AHI: acute hemodynamic index. Model 0 included Chagas disease as heart failure etiology; chronic kidney disease; history of cancer; and home use of beta-blockers, loop and thiazide diuretics, digitalis, and spironolactone. Models 1 to 5 included all variables from model 0 plus another parameter and cut-off value, as follows: Model 1: heart rate ≤ 88 bpm; Model 2: systolic blood pressure ≤ 100 mmHg; Model 3: systolic blood pressure ≤ 120 mmHg; Model 4: diastolic blood pressure ≤ 60 mmHg; Model 5: AHI ≤ 4 mmHg⋅bpm.*

**Figure 2:** – Odds ratios according to a multivariate regression model including heart failure etiology, comorbidities, medication use, and acute hemodynamic index (AHI) of patients admitted with acute decompensated heart failure with reduced ejection fraction (n = 463).

**Figure 3:** – In-hospital mortality indices of patients with acute decompensated heart failure with reduced ejection fraction according to the presence of prognostic factors. *p < 0.05 in comparison to “No” within the same prognostic parameter.

## Discussion

The present study introduced the AHI and demonstrated that it is an independent predictor of in-hospital mortality in patients with acute decompensated HFrEF. In-hospital mortality in patients with acute decompensated HF is high, as shown by this Brazilian registry and by studies conducted in other countries.^27^Different reasons for this high short-term mortality include age, comorbidities, and the delay between symptom onset and hospital admission.^27^Since the management of patients with acute HF may include invasive and high-cost procedures such as circulatory support, it is critical to validate prognostic factors that can help guiding therapeutic decisions.^28^

Acute decompensated HF can be managed by HF specialists, general cardiologists, intensivists, emergency physicians, or internists; this can be performed in emergency departments, hospital wards, or intensive care units.^2^The physician’s experience and the available resources can vary substantially. Together with the patients’ diversity, these aspects hinder the production of widely applicable prognostic scores. Despite the recent attention received by biomarkers,^29^for example, their verification may not be available in remote or low-income health facilities. Nohria et al.^6^have introduced a practical clinical approach for categorizing patients with hemodynamic profiles, thus enabling prognosis prediction and guiding treatment in acute HF settings. This approach relies on clinician experience^8 , 9^and may be less useful when considering non-HF specialists. Our results corroborate the lack of accuracy of cardiovascular physical examinations,^9^since 11% of the patients were classified as hemodynamic profile A despite having acute decompensated HF.

Heart rate and blood pressure measurements are available in virtually any healthcare facility with good accuracy and requiring minimal training.^11 , 12^Previous studies have tried to use blood pressure and heart rate as prognostic factors in acute decompensated HF; the relationship between heart rate and prognosis in heart disease has been known for decades. Since the emergence of therapies using beta-blockers and more recently, ivabradine, low heart rates have been considered a target in the treatment of stable HF.^19^On the other hand, chronotropic incompetence is also a risk marker. Patients whose heart rates do not increase during exercise have worst prognoses than those with normal heart rate reserves, even with the use of beta-blockers.^20 , 21^Although previous studies have determined the expected increase in heart rate during an exercise test,^20 , 21^no normality values have been established for heart rate increases during “fight or flight” situations such as the admission to emergency rooms. Japanese patients with acute decompensated HF admitted with heart rates above 120 bpm presented lower mortality indices than those with lower heart rates.^15^Conversely, high heart rate was considered an independent predictor of short-term mortality in patients with acute decompensated HF in other studies.^16 , 30 , 31^

The OPTIMIZE-HF^14^registry found that systolic blood pressure values below 120 mmHg characterized patients with acute decompensated HF who had poor prognoses despite medical therapy. Low systolic blood pressure levels also indicated high short-term risk in a European cohort.^13^In our study, blood pressure below 120 mmHg was not independently related to mortality in a multivariate analysis. Patients in the BREATHE registry were younger, and treatment protocols were more updated when compared to those used in studies conducted almost a decade earlier. Furthermore, both studies^13 , 14^included patients with preserved and reduced ejection fraction, and the prognostic value of blood pressure is known to vary according to the left ventricular ejection fraction.^32^Low pulse pressure was defined as an independent predictor of mortality in acute decompensate HF by the VMAC-HF study.^33^Since the publication of this trial, HF therapy has evolved substantially, which may explain the lack of prognostic power of pulse pressure in our patients.

The intrinsic interaction between blood pressure and heart rates and how they are affected by HF medications may have influenced the results of previous investigations on each of these parameters. To our knowledge, this is the first study to introduce an index that analyzes both heart rate and pulse pressure in patients with acute decompensated HF; moreover, we have shown that the prognostic ability of the AHI is higher than that of heart rate or blood pressure alone.

### Limitations

The present analysis has limitations. First, in-hospital mortality was based on investigator reports instead of being adjudicated. In fact, registries are observational studies and analyzing the treatment delivered to each patient was not within the scope of our study. As our main objective was to analyze the usefulness of an easily obtained index to be applied as soon as patients arrive in the emergency room, and considering the unavailability of troponin and brain natriuretic peptide (BNP) tests in some Brazilian health facilities, laboratory parameters were not included in the model.

Data in the registry was not obtained by any specific protocol, and blood pressure and heart rate measurements may have been performed using different equipment. Nevertheless, blood pressure and heart rate are vital signs that require minimal training for their measurement.^11 , 12^Additionally, the fact that the registry had no standardized assessment methods enhances the clinical applicability of our study, as it shows realistic results.

The present results are restricted to patients with HFrEF. The study was conducted from 2011 to 2012, before the approval of new HF medications as ivabradine and salcubitril-valsartan,^19^which could influence AHI values.

The Brazilian population is very diverse regarding ethnicity and access to health care facilities. The study included private and public hospitals in all regions of the country.^23^Although the generalization to other populations may be limited, we highlight that the demographical and clinical data of patients included in this registry are very similar to those of other cohorts.^14 , 16 , 30 , 31^

Finally, the AUC in the ROC analysis of the AHI was relatively low. Nevertheless, its sensitivity was quite good and this may be useful to guide emergency physicians while triaging patients.

## Conclusion

Different prognostic factors have been proposed in acute decompensated HF but rely on biomarker measurement, medical staff training, and technology; these may not be widely available. The AHI is a practical, objective, and easily obtained prognostic factor for in-hospital mortality in patients with acute decompensated HF. Further prospective studies should evaluate the reproducibility of these results in other populations.
